# Brain morphology normative modelling platform for abnormality and centile estimation: Brain MoNoCle

**DOI:** 10.1162/imag_a_00438

**Published:** 2025-01-10

**Authors:** Bethany Little, Nida Alyas, Alexander Surtees, Gavin P Winston, John S Duncan, David A Cousins, John-Paul Taylor, Peter Taylor, Karoline Leiberg, Yujiang Wang

**Affiliations:** CNNP Lab ( www.cnnp-lab.com ), School of Computing, Newcastle University, Newcastle upon Tyne, United Kingdom; Faculty of Medical Sciences, Newcastle University, Newcastle upon Tyne, United Kingdom; Research Software Engineers, Newcastle University, Newcastle-upon-Tyne, United Kingdom; UCL Queen Square Institute of Neurology, Queen Square, London, United Kingdom; Department of Medicine (Division of Neurology), Queen’s University, Kingston, Canada; Cumbria, Northumberland Tyne and Wear NHS Foundation Trust, Newcastle upon Tyne, United Kingdom

**Keywords:** normative model, brain structure, structural abnormality, morphology, bipolar disorder, temporal lobe epilepsy

## Abstract

Normative models of brain structure estimate the effects of covariates such as age and sex using large samples of healthy controls. These models can then be applied to, for example, smaller clinical cohorts to distinguish disease effects from other covariates. However, these advanced statistical modelling approaches can be difficult to access, and processing large healthy cohorts is computationally demanding. Thus, accessible platforms with pre-trained normative models are needed. We present such a platform for brain morphology analysis as an open-source web applicationhttps://cnnplab.shinyapps.io/BrainMoNoCle/, with six key features: (i) user-friendly web interface, (ii) individual and group outputs, (iii) multi-site analysis, (iv) regional and whole-brain analysis, (v) integration with existing tools, and (vi) featuring multiple morphology metrics. Using a diverse sample of 3,276 healthy controls across 21 sites, we pre-trained normative models on various metrics. We validated the models with a small sample of individuals with bipolar disorder, showing outputs that aligned closely with existing literature only after applying our normative modelling. Using a cohort of people with temporal lobe epilepsy, we showed that individual-level abnormalities were in line with seizure lateralisation. Finally, with the ability to investigate multiple morphology measures in the same framework, we found that biological covariates are better explained in specific morphology measures, and for applications, only some measures are sensitive to the disease process. Our platform offers a comprehensive framework to analyse brain morphology in clinical and research settings. Validations confirm the superiority of normative models and the advantage of investigating a range of brain morphology metrics together.

## 1 Introduction

Brain morphology, the study of the shape and size of brain structures, can be used to track healthy brain development and detect abnormalities associated with underlying disease processes. Normative modelling of brain morphology uses large and diverse datasets to estimate healthy variance across the lifespan. In neuroscience research, normative models can reliably remove biological and technical covariates from unseen data without removing, for example, disease effects (Pomponio et al., 2020), which is especially valuable for small samples with a limited number of control subjects. Further, the ability of normative modelling to estimate abnormalities in individuals is crucial for clinical applications, as it enables systematic biomarker discovery and supports translational uses in diagnosis, stratification, and localisation (Little et al., 2024;Loreto et al., 2024;P. N. Taylor et al., 2022). Therefore, normative modelling of brain morphology is an indispensable framework that should be available and accessible to all researchers.

To enable researchers without high levels of technical/statistical know-how, or resources for time and computationally demanding tasks, to benefit from the power of the normative modelling framework, a freely available, pre-trained modelling platform is needed. The normative models should be based on large, diverse, healthy population data, and be easily applied to new data. Recent efforts in this field include several open-source tools, some allowing users to upload new, unseen brain morphology data to a web interface and generate individual abnormality scores (e.g. z-scores or centiles) (Bethlehem et al., 2021;Ge et al., 2024;Rutherford et al., 2023). Each of these tools has some advantages: for example, (i) being accessible as an online tool that can be easily used without any need for software download/installation or writing/running scripts, (ii) providing individual and group-level outputs, (iii) multi-site data—as often seen in neuroscience research—can be analysed, (iv) analysing brain shape on whole hemispheres and smaller regions, (v) seamless integration with existing neuroimaging software such as FreeSurfer, and (vi) the option to explore a variety of metrics.

We present a normative modelling tool of brain morphology that combines all six key features in one open and web-based application: Brain MoNoCle (Brain Morphology Normative modelling platform for abnormality and Centile estimation). We included a large and diverse sample of 3,276 healthy controls across 21 sites to pre-train normative models in a variety of brain morphology measures that comprehensively quantify cortical shape, including three novel metrics that were only recently proposed (Wang et al., 2021). As a first validation, we demonstrate how normative modelling improves reliability and reproducibility in a small clinical dataset of individuals with bipolar disorder (BD). We further validate our outputs in a dataset of temporal lobe epilepsy (TLE) at the group level, and illustrate how individual patient abnormality scores agree with their seizure lateralisation. Finally, with the option to explore a variety of morphology metrics on our platform, we highlight the importance of investigating multiple metrics at the same time both for normative modelling itself and for clinical applications.

## 2 Materials and Methods

### 2.1 Normative data

We collated 3T T1-weighted MRI scans from 3,276 healthy individuals from several large public and in-house datasets, detailed inSupplementary Materials S1(Greene et al., 2018;Himmelberg et al., 2023;LaMontagne et al., 2019;Nigg et al., 2023;Nooner et al., 2012;Nugent et al., 2022;Shafto et al., 2014;J. R. Taylor et al., 2017;Van Essen et al., 2013;Zareba et al., 2022). The age in the total dataset ranged from 5 to 95 years old; the age range and sex distribution for each study are illustrated inFigure 1. Scanning protocols differed across, and sometimes within datasets, which we corrected statistically in a later step. All studies had ethical approval from relevant institutional ethics boards and included written consent from participants. We present here the initial dataset included in v1.0 of our app; however, we aim to continuously add to our normative reference dataset. Users of our web platform should, therefore, check the latest summary of the dataset shown in the app when reporting their own results.

**Fig. 1.**
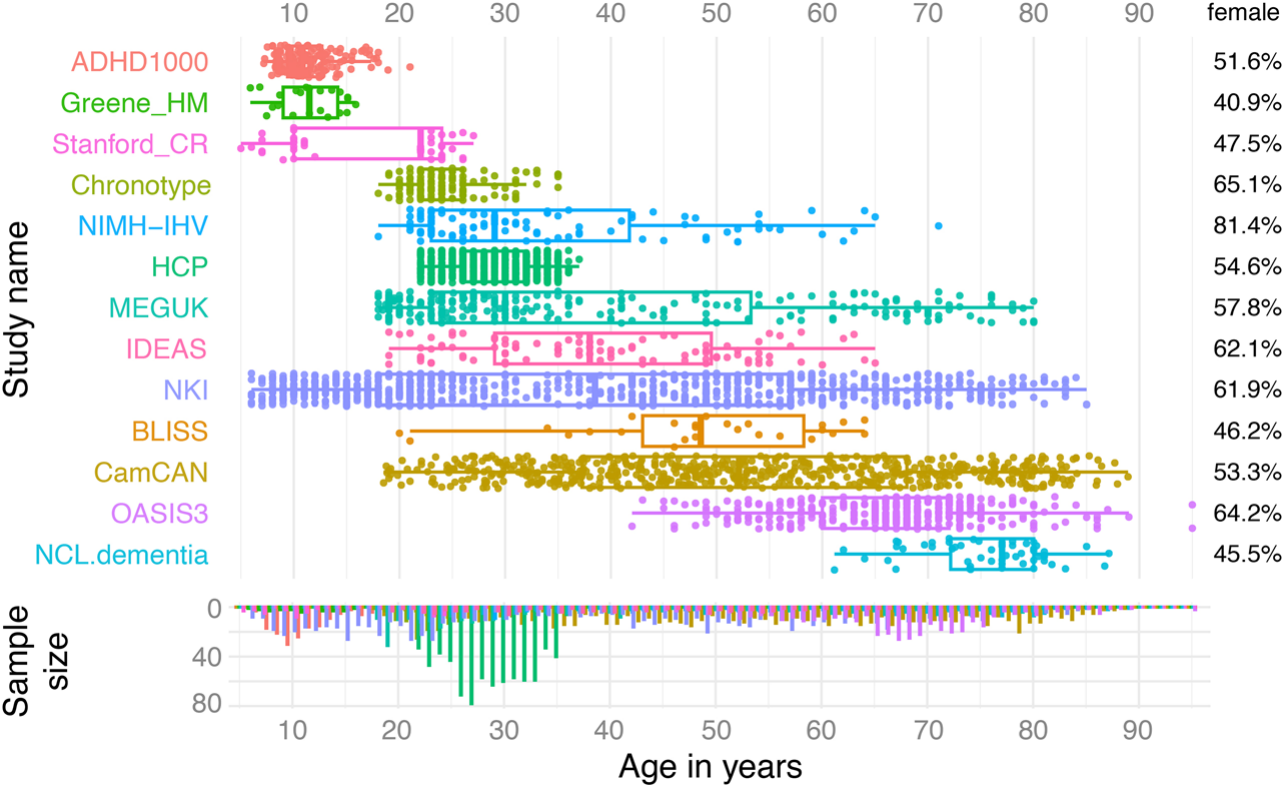
Demographics of the data underlying the normative model. Age distributions and proportion of female participants are shown for each study. Some studies contained multi-site data (e.g. MEGUK), but these are not shown separately here.

### 2.2 Pre-processing

T1-weighted MRI scans were pre-processed in FreeSurfer (Fischl, 2012) using the standard*recon-all*pipeline, which includes removal of non-brain tissue, segmentation of grey and white matter surfaces, and cortical parcellation. We also ran the localGI pipeline (Schaer et al., 2012) to yield smoothed outer pial surfaces. The*aparcstats2table*command was used to generate measures of cortical thickness, cortical volume, and pial surface area, for 68 brain regions according to the Desikan–Killiany parcellation atlas (Desikan et al., 2006). The version of FreeSurfer varied across datasets (seeSupplementary Materials S1), which was corrected during site/batch harmonisation.

### 2.3 Cortical morphology measures

The traditional morphological measures of cortical thickness, pial surface area, and exposed surface area are known to covary (Wang et al., 2016). Failure to account for this covariance can lose and confuse information about the complex, folded shape of the brain. A recently developed framework proposed a universal scaling law of cortical folding that accounts for covariance between cortical thickness, pial surface area, and exposed surface area (Mota & Herculano-Houzel, 2015). From this scaling law, three biologically interpretable independent components, K, I, and S, can be derived for hemispheres, lobes, and individual regions (Leiberg et al., 2021;Wang et al., 2016,^2019^,^2021^). The dimensionless measure K reflects tension acting on the cortex and is relatively preserved across species, but appears to be sensitive to ageing and disease processes (Leiberg et al., 2021;Wang et al., 2019,^2021^). Isometric term I is orthogonal and statistically independent to K and captures information about isometric size. S is a cross-product of K and I that captures all remaining information about shape, reflecting complexity of cortical folding. For example, if a cortical structure is isometrically rescaled in all dimensions, it changes I, but not K or S. K, I, and S are orthogonal and statistically independent to each other.

As an example, in TLE, these components captured structural changes that were not detected with traditional metrics (Wang et al., 2021). K, I, and S, therefore, offer a novel re-conceptualisation of brain morphology measures that can detect nuanced morphological abnormalities. We used the toolbox developed byWang et al. (2021)(https://github.com/cnnp-lab/CorticalFoldingAnalysisTools) to calculate K, I, and S for each hemisphere.

### 2.4 Quality control

Some of the public datasets included quality control steps as part of the study design, which are reported in the original study publications. We detected outliers in the entire dataset statistically: we ran our*gamlss*model described below for each structural metric for each region, and flagged outliers defined by residuals more than five median absolute deviations. In addition, we also detected outliers based on visual inspections of plots: for each dataset, we plotted each brain metric at the hemisphere level against age to flag outliers within each dataset. These were then cross checked with outliers that were detected statistically. We excluded participants who were flagged as an outlier in any of these models; we performed listwise deletion rather than pairwise deletion so that the same normative reference dataset was used for each normative model, allowing comparisons of model statistics across models.

### 2.5 Exemplar clinical datasets

To demonstrate the utility of our normative models in predicting abnormalities in patient groups and individuals, we included two exemplar clinical datasets. A sample of 133 adults with mesial TLE (mTLE; n = 74 right hemisphere seizure onset; n = 59 left hemisphere seizure onset) and 99 healthy controls (HC-mTLE) were acquired from the recent IDEAS dataset release (P. N. Taylor et al., 2024), which was approved by a Research Ethics Committee (22/SC/0016). We also included a sample of 56 adults with bipolar disorder (BD) and 26 healthy controls (HC-BD) from the Bipolar Lithium Imaging and Spectroscopy Study (BLISS) (Little et al., 2024;Smith et al., 2018). The study was granted a favourable ethical opinion by a United Kingdom National Research Ethics Committee (14/NE/1135), and all participants provided written informed consent.

Descriptive statistics for these datasets are summarised inTable 1. See the respective publications for full details of each sample and neuroimaging pre-processing steps.

**Table 1.** Demographics of two clinical datasets.

	BD	HC-BD	mTLE left	mTLE right	HC-mTLE
N	56	26	74	59	99
Age [mean (SD)]	45.36 (12.15)	48.46 (11.80)	36.0 (11.2)	38.2 (10.8)	39.1 (12.1)
Sex [n female (%)]	35 (62%)	12 (46%)	43 (58%)	39 (66%)	62 (63%)

SD = standard deviation; HC = healthy controls; BD = bipolar disorder; mTLE = mesial temporal lobe epilepsy.

### 2.6 Software structure

We designed our software as an R Shiny App, and it includes three aspects: (i) Pre-training normative models for each morphological measure, which can be readily used without re-fitting statistical models to normative data. (ii) At the back-end of the R Shiny App, computing z-scores and centiles for new unseen data based on the pre-trained models. (iii) At the front-end of the R Shiny App, providing the user with an intuitive interface that accepts outputs directly from existing neuroimaging software, such as FreeSurfer. Each aspect is summarised below, and more technical details can be found in theSupplementary Materials.

#### 2.6.1 Pre-training normative models

All brain metrics from the normative data were log-transformed before being used to train the normative models, so different metrics measuring different dimensionalities (e.g. thickness*vs.*surface area) can be treated in the same way for the normative model. Generalized additive models for location scale and shape (GAMLSS) using the gamlss package (https://cran.r-project.org/web/packages/gamlss/) were used to simultaneously model the parameters (mean, standard deviation, skew, and kurtosis) of the distribution as response variables of the explanatory variables sex, age, and scanning site/batch. Specifically, in our model, the mean depends on sex (fixed effect), site (random effect), and a smooth function of age; the standard deviation depends on sex (fixed effect), site (random effect), and a smooth function of age; the skew depends on sex (fixed effect) and a smooth function of age; and the kurtosis depends on a smooth function of age. See theSupplementary Materials S2for a more detailed description and justification of the statistical model. Normative models were fitted independently for each region (from the Desikan–Killiany atlas) and hemisphere, and each morphometric measure. Residuals were retained for later visualisations.

#### 2.6.2 Predicting abnormalities in unseen data

The pre-trained normative models are implemented on the R Shiny App back-end to score new, unseen individuals. A healthy control (HC) cohort from the new unseen site/batch is currently required.

First, we predicted the distribution parameters based on the HCs in each new site/batch and calculated residuals relative to one of the normative scanning sites. The mean of the residuals from the HCs in the unseen data was then used to calculate the site-specific offset needed to harmonise the unseen dataset with the normative data.

To obtain z-score, we then calculated the residuals for each individual in the new unseen data relative to their site mean and divided by a standard deviation. The latter is calculated as follows: if there are less than 30 HCs, we used the average standard deviation seen across normative data sites/batches in the pre-trained normative models. We estimated the standard deviation from the unseen HCs only if there were 30 or more HCs in the dataset. This approach ensures accurate estimations of new sites’ standard deviations, as a sample size of 30 provides a 55% probability of being within 10% accuracy and a 95% probability of being within 25% accuracy (Schillaci & Schillaci, 2022).

The site-specific mean, (site-specific) standard deviation, skew, and kurtosis from the pre-trained normative model were used to calculate centiles. See theSupplementary Materials S2for a detailed description of the statistical pipeline.

#### 2.6.3 Using the Brain MoNoCle web user interface

To run our pipeline to predict abnormalities in unseen data as described above, we used our web platform Brain MoNoCle. Users can follow the same steps to run the pipeline on their own data.

First, we uploaded pre-processed brain imaging data tables. For traditional brain imaging metrics, data should be pre-processed using FreeSurfer (e.g., the standard*recon-all*command) and the structural metrics for each hemisphere should be exported as csv files using the*aparcstats2table*command; then the csv file for each hemisphere can be directly uploaded to our web interface. For morphology measures of independent components, the data tables from our cortical folding toolbox (https://github.com/cnnp-lab/CorticalFoldingAnalysisTools) can be directly uploaded. We also uploaded meta-data in a csv file containing subject IDs, age, sex, group, dataset, scanning site, and session. After selecting “Run Model” to start the analysis, z-scores, group summary statistics, and centiles are available to view and download as csv files, using the tabs in the main panel. Users can export plots by selecting the “Brain plot” and “Scatter plot” tabs. A html report is available to download using the “Report” tab.

### 2.7 Statistical analysis

All statistical analyses were performed in R Studio v4.3.2. Each test and associated sample size are stated in the results section.

## 3 Results

### 3.1 Pre-trained normative models on web platform

To pre-train normative models, we used data from 3,276 healthy individuals from several large public and in-house datasets, detailed inSupplementary Materials S1(Greene et al., 2018;Himmelberg et al., 2023;LaMontagne et al., 2019;Nigg et al., 2023;Nooner et al., 2012;Nugent et al., 2022;Shafto et al., 2014;J. R. Taylor et al., 2017;Van Essen et al., 2013;Zareba et al., 2022). We focused on including a variety of scanning protocols/sites (total 21 sites) to enable mixed-effect modelling, and we achieved a larger overall sample size than recommended by previous normative models (Ge et al., 2024). We performed a subsampling analysis that showed our models stabilised, seeSupplementary Materials S3for details. The age in the total normative dataset ranged from 5 to 95 years old and is shown by data source inFigure 1. With these data, we pre-trained our normative models for the whole hemisphere, each brain region, and morphology metric (seeSection 2for details of statistical models). We incorporated these pre-trained normative models on a web platform (Brain MoNoCle) to allow users to upload their own datasets to find morphological abnormalities in individuals and groups. In the following, we will validate our normative modelling framework and web platform outputs in a variety of ways, and demonstrate some biological insight.

### 3.2 Cortical thickness abnormalities in a sample of patients with bipolar disorder more closely match previous findings when using the normative model

**Fig. 2.**
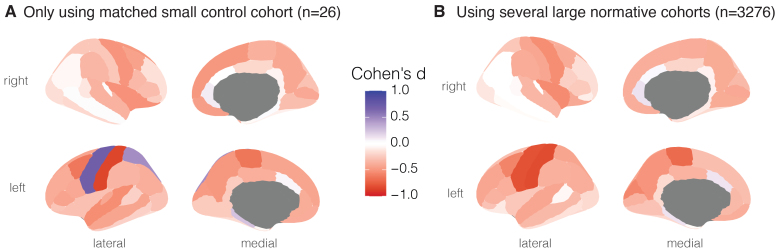
Alterations in cortical thickness associated with bipolar disorder derived from a case–control study*vs.*normative modelling. Group-level abnormalities in cortical thickness in n = 56 people with bipolar disorder for (A) a small, matched control group (n = 26) and (B) the normative reference population (n = 3,276).

To validate the normative modelling framework and outputs, we first investigated the group-level differences in a small, well-defined clinical cohort of bipolar disorder (BD, n = 56) and matched controls (BD-HC, n = 26). We specifically wanted to see the difference in outputs between using a traditional case–control comparison approach only using the matched controls (Fig. 2A)*vs.*using our normative model instead (Fig. 2B). When using the small BD-HC group, effect sizes (Cohen’s d) suggested that cortical thinning was greatest in the left post-central gyrus (d = -0.85) and that the cortex was thicker in BD in the left pre-central gyrus (d = 0.70). However, when using the normative model pipeline, the same sample showed similar thinning in the left post-central gyrus (d = -0.83), but cortical*thinning*in the left pre-central gyrus (d = -0.8). The latter of these findings, obtained through normative modelling, is more in line with previous findings from a large sample ENIGMA study showing cortical thinning across the cortex (Hibar et al., 2018).

### 3.3 Group-level cortical thickness abnormalities in mesial temporal lobe epilepsy agree with previous findings

**Fig. 3.**
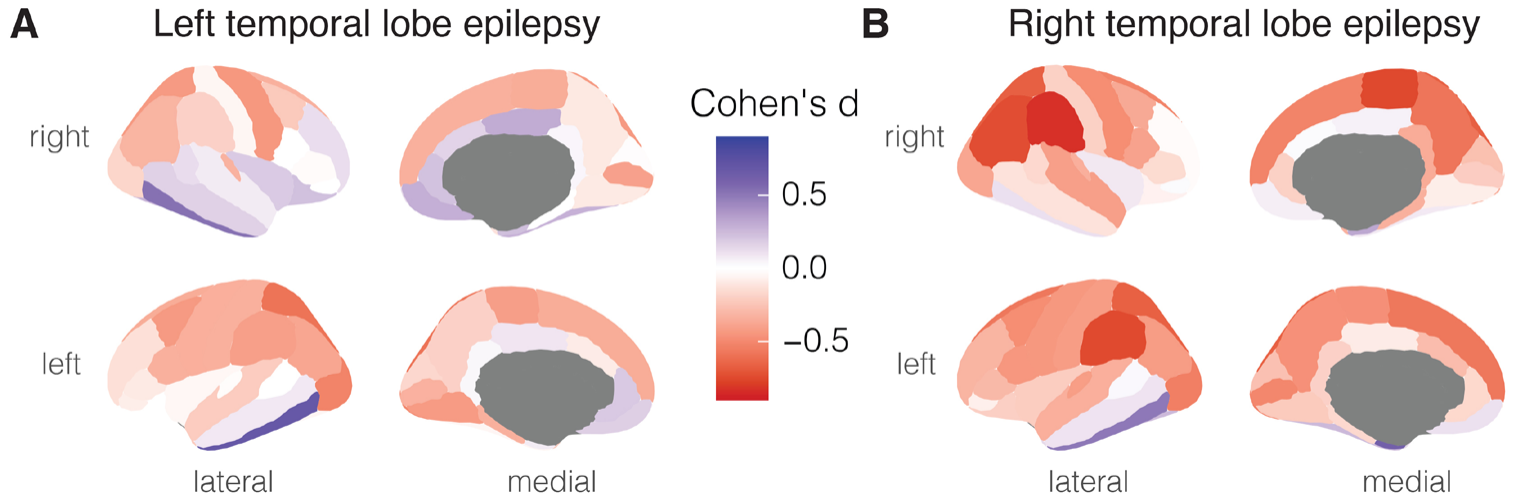
Group-level output for mesial temporal lobe epilepsy cohort after normative modelling. Group-level summary of abnormalities in cortical thickness for left mTLE (n = 74, A) and right mTLE (n = 59, B), showing Cohen’s d effect size for each cortical region.

We validated our normative model outputs in a large sample of individuals with mTLE (74 left mTLE, 59 right mTLE) and matched controls (n = 99) (P. N. Taylor et al., 2024).Figure 3shows cortical thickness abnormality estimates for right mTLE and left mTLE groups. We found widespread cortical thinning, especially in the right mTLE group, in cortical regions including the precentral gyrus, supramarginal gyrus, and inferior parietal gyrus. This result reproduces both the findings from the IDEAS and ENIGMA-epilepsy studies. We quantitatively assessed this by correlating the effect sizes for each brain region generated by Brain MoNoCle in our mTLE sample with the effect sizes reported in the ENIGMA study (Whelan et al., 2018); results showed agreement between both sets of effect sizes (seeSupplementary Materials S4.1).

As a supplementary step, we compared the z-scores for each brain region produced by Brain MoNoCle with z-scores produced by a similar normative modelling platform, CentileBrain, using the IDEAS healthy control group (n = 99). Results show good agreement between both apps (correlation larger than 0.75 in approx. 80% of brain regions, seeSupplementary Materials S5).

### 3.4 Individual-level abnormalities in certain measures agree with clinical lateralisation of seizure onset

**Fig. 4.**
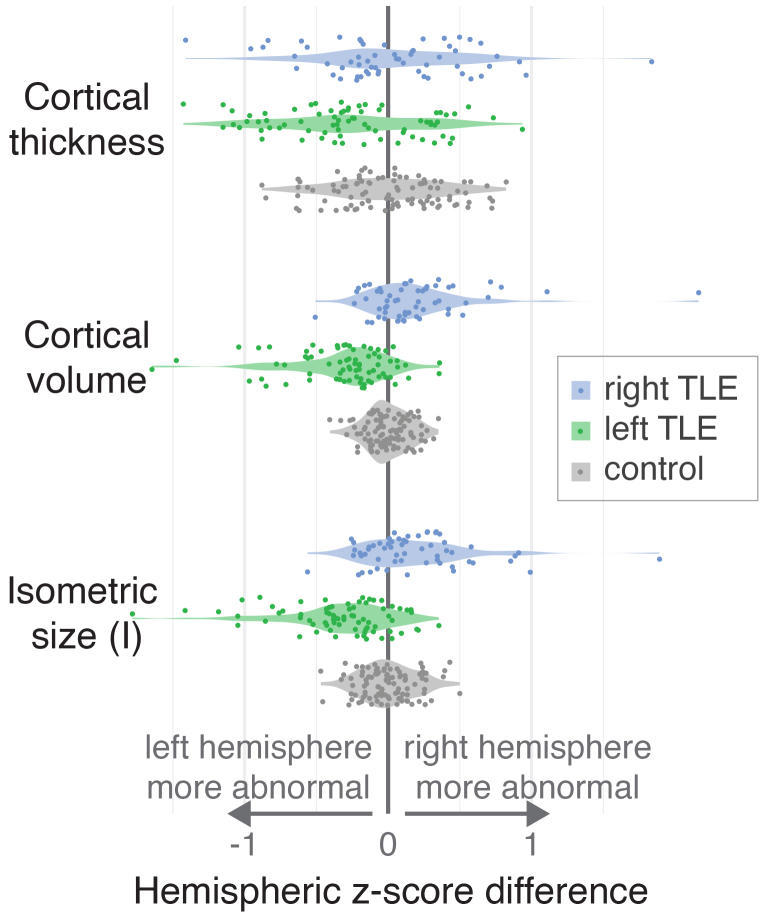
Individual-level z-scores after normative modelling for mTLE cohort. Difference in hemisphere-level z-score between left and right hemisphere is shown in controls and right/left TLE subgroups for three example morphological measures. Individual subjects are shown as single data points, distributions of subjects are displayed as violin plots.

To validate individual-level outputs and abnormalities, we used seizure lateralisation from the IDEAS dataset (P. N. Taylor et al., 2024). For each subject, we extracted the z-score difference between left and right hemispheres in cortical thickness and other metrics (Fig. 4). Controls, as expected, had a distribution around zero in all measures after regressing out healthy biological covariates.

The measure most frequently used in cortical morphometry, cortical thickness, did not lateralise individual patients well, and most patient z-scores differences were within the same range and distribution as the controls.

Given that our normative modelling platform offers the ability to analyse multiple morphology measures, and given that cortical thickness is known to covary with other measures (such as surface area and volume), we investigated all metrics implemented on the platform. This included three statistically independent novel measures. We demonstrated that both cortical volume and I—our novel morphometric for isometric size—were best at lateralising at the hemisphere level (Fig. 4). Specifically, for cortical volume, most (78.2%) patients had a z-score difference greater/smaller than zero, indicating lateralisation in agreement with clinical metadata. For I, 75.9% of patients showed the correct laterality. Further, 36.1% of patients were outside of 2 standard deviations of the control for cortical volume, and 32.3% for I. Confusion matrices showing predictive performance for lateralisation using the sign of the z-score difference between left and right hemisphere can be found in theSupplementary Materials S4.2. Overall performance accuracy was larger than 0.75 for both volume and I.

### 3.5 Covariates explain more variance in independent component K than in other structural MRI measures

Given the observed specificity in particular measures for seizure lateralisation, we explored the differences between morphological measures further to establish a baseline for future applications. To this end, we investigated the normative models accounting for age, sex and scanning site for each measure.

**Fig. 5.**
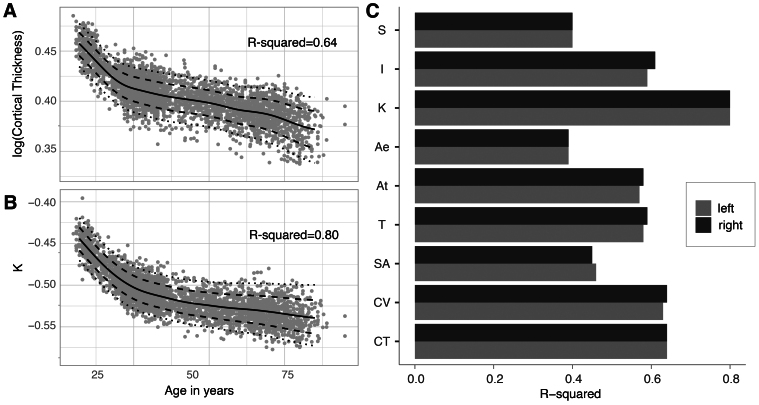
Variance explained by normative model in each morphometric. (A & B) Harmonised normative data (grey dots) and predicted model centiles of mean cortical thickness and K across the lifespan (n = 3,276). (C) Model fit statistics$R^{2}$for each metric and hemisphere. CT, CV, and SA are structural metrics estimated using FreeSurfer; T, At, Ae, K, I, and S are structural metrics estimated using the Cortical Folding toolbox. CT = cortical thickness, CV = cortical volume, SA = surface area, T = average thickness, At = total pial surface area, Ae = exposed surface area.

Figure 5A and Bshows the fitted normative model over age for two example measures: cortical thickness and K—a novel independent morphometric that is known to change with age (Wang et al., 2021). Both thickness and K decrease over age with steeper declines in early and later life.

To compare morphology measures more directly, we obtained the$R^{2}$of the normative model fit for each measure,Figure 5C. All measures used the same statistical model formulas of age, sex, and site. Out of all the measures implemented, K shows the best model fit ($R^{2}=0.8$for both left and right hemisphere), superior to all other metrics with$R^{2}$around or below 0.6.

## 4 Discussion

### 4.1 Summary

Brain MoNoCle is a user-friendly online normative modelling platform for brain morphology analysis. The platform combines and unifies the most frequently requested and desired features of existing approaches and toolboxes in one, importantly including the option to analyse multiple morphology measures under one framework. We validated our normative models and platform outputs in clinical cohorts through a series of tests, including replicating previous findings from ENIGMA studies. We also provided an individual-level output validation in a sample of mTLE by demonstrating agreement of our outputs with the clinical seizure lateralisation. Particularly, we highlighted that both biological covariates, as well as disease processes, are uniquely expressed in different morphological measures. This implies that brain normative modelling should be performed in a range of measures to be useful for brain morphology analysis in health and disease.

### 4.2 Validations

We demonstrated how our pipeline can be applied to clinical datasets and what outputs can be obtained in a sample of people with BD, and a sample of people with mTLE. Compared with the traditional case–control pipeline, in which BD patients were compared with matched healthy controls, our normative pipeline yielded abnormality estimates that were more in line with previous research, for example, a large-scale ENIGMA study of cortical thickness in BD, despite a relatively small patient sample (Hibar et al., 2018). In mTLE, we showed results similar to a recent report of the data release (P. N. Taylor et al., 2024). In particular, we did not find large abnormalities in mTLE patients; this may be because we only show cortical data and mTLE are associated with structural changes in subcortical regions, such as the hippocampus (Whelan et al., 2018). We also tested for lateralisation of the hemispheric abnormalities, and found a generally good agreement with clinical metadata, despite only using cortical data. The reported effect sizes compared with controls are in line with previous reports of lateralisation using cortical information only (Pustina et al., 2015;Whelan et al., 2018). We conclude that our normative models provide reasonable outputs in small and large samples, and that individual-level outputs are also in line with expectations. We hope Brain MoNoCle will be helpful in future analysis of cortical morphology.

### 4.3 Normative sample

We trained our models using healthy control data from several large databases (Supplementary Materials S1 and S6), similar to other normative modelling approaches. Our model is stable with the current sample size of 3,276 subjects. However, in mixed effect modelling, the number of levels in the random effect (i.e., the number of sites) is also critical. It is recommended to use a fixed effect if there are fewer than 10 levels, and to apply caution with 20 levels as a random effect. We included 21 sites, and suggest that future work should prioritize increasing the number of sites in normative datasets, with diverse global representation, rather than focusing solely on total subject count.

### 4.4 Methodological advance

Our normative models and web platform are an addition to existing free toolboxes for modelling cortical morphometry (Attyé et al., 2024;Bethlehem et al., 2021;Ge et al., 2024;Manjón & Coupé, 2016;Rutherford et al., 2023). Brain MoNoCle, however, differs to all of these in some ways, including no requirement for any coding or running scripts from the user, no need to download software, outputs include group-level analysis compared with controls as well as individual abnormalities as z-scores and centiles, analysis is on full hemispheres and regions, and outputs are visualised in plots and available as tables of z-scores and centiles. There are differences in the underlying models as well. For example, our model harmonises scanning site effects for mean and variance, avoiding separate steps as found in Combat (Johnson et al., 2007), which makes separate assumptions. Specifically, Combat assumes that after removing covariate effects on the mean, the data are normally distributed, and site effects on mean and variance are estimated based on this assumption. However, this is often not the case, since data may follow a non-normal distribution with higher distribution parameters of skew and kurtosis also depending on covariates. Further, in our model, information is pooled from the male and female populations to directly estimate sex covariate effects, and we use flexible smooth terms for age effects and explicitly model skew and kurtosis with GAMLSS. Lastly, our web platform includes normative models of a range of metrics, including traditional measures such as thickness, volume, and pial surface area, but also independent morphometrics which account for the covariance of those measures.

### 4.5 New biological insight

Through our exploration of multiple cortical morphometrics, we were able to compare normative models for traditional measures, such as cortical thickness and surface area, but also novel statistically independent morphometrics. We found that one of these novel morphometrics “K” (also termed “tension component”) displayed a far superior performance as a normative model of age and sex with$R^{2}=0.8$compared with other morphometrics that achieve$R^{2}$between 0.4 and 0.6. This observation has two implications: firstly, there might be better morphometrics to use to model age and sex effects, and extract disease-specific effects, as alluded to in the first paper proposing “K” as a novel morphometric (Wang et al., 2021). Secondly, traditional morphometrics clearly have residual unexplained variance due to their statistical interdependence. This implies that investigations of measures such as cortical thickness and surface area should consider their covariance, rather than interpreting them in isolation. The cortex, as a biological structure obeying physical constraints, clearly does not have independent processes to develop its thickness*vs*. surface area*vs*. overall size. Our platform, offering analysis streams for all traditional morphometrics and novel morphometrics, therefore, serves as a starting point for future statistically robust analyses of brain morphology.

### 4.6 Roadmap for future development

We have three concrete developments planned for our normative modelling platform. First, we will incorporate recently proposed multiscale morphometrics (Chen et al., 2022;Leiberg et al., 2023;Wang et al., 2023) to allow users to access the most recent cutting-edge developments in morphological analysis. Second, we currently use one Freesurfer parcellation of the brain to analyse finer regions. We plan to incorporate more atlases, and in the same step, incorporate the possibility to jointly model related regions (e.g. neighbouring regions) to increase robustness of the model. We also note that vertex-wise data and surface-based statistics would be a useful addition. Third, with the increasing availability of longitudinal data, we plan to extend our normative model to accept multi-session longitudinal clinical datasets and statistically account for these adequately (see e.g.Bučková et al. (2023)for some suggestions). Further, we will add more normative data from diverse geographical areas. We will also implement analysis capacity to compare morphology measures more directly on the web platform. Finally, we aim to add more structural metrics such as subcortical volumes, and integrate output from other neuroimaging tools, such as CIVET and volBrain (Lee et al., 2006;Manjón & Coupé, 2016).

## Supplementary Material

Supplementary Material

## Data Availability

Normative data may be available at the discretion of the data holders, please see the website of individual datasets for more information. The subset of the IDEAS mesial TLE dataset is freely available with the associated paper (P. N. Taylor et al., 2024).
