# Supplementary material for: Brain morphology normative modelling platform for abnormality and centile estimation: Brain MoNoCle
Source: Imaging Neurosci (Camb). 2025 Jan 10;3:imag_a_00438. doi: 10.1162/imag_a_00438 (PMC12319752; doi:10.1162/imag_a_00438)
Supplement: Supplementary Material [file imag_a_00438-supp.pdf]

Supplementary Materials

S1 Normative data

Dataset (site)	Sample size [n]	Age in years [median(range)]	Sex [n female (%)]	Location [Country]	FreeSurfer [Version]
ADHD1000	153	11 (7 - 21)	79 (51.6%)	USA	7.3.2
BLISS	26	49 (20 - 64)	12 (46.2%)	UK	6.0.1
CamCAN (s1)	495	50 (19 - 89)	259 (52.3%)	UK	5.3.0
CamCAN (s2)	83	59 (19 - 86)	49 (59.0%)	UK	5.3.0
Chronotype	126	24 (18 - 35)	82 (65.1%)	Poland	7.3.2
Greene-HM	22	11 (6 - 16)	9 (40.9%)	USA	7.3.2
HCP	678	29 (22 - 37)	370 (54.6%)	USA	5.2
MEGUK (Aston1)	29	23 (18 - 49)	25 (86.2%)	UK	7.3.2
MEGUK (Aston2)	69	33 (18 - 63)	39 (56.5%)	UK	7.3.2
MEGUK (Cambridge)	71	42 (19 - 80)	37 (52.1%)	UK	7.3.2
MEGUK (Glasgow)	24	26 (18 - 34)	13 (54.2%)	UK	7.3.2
MEGUK (Oxford)	63	40 (20 - 80)	34 (54.0%)	UK	7.3.2
NCL-dementia (s1)	29	77 (62 - 85)	11 (37.9%)	UK	6.0.1
NCL-dementia (s2)	26	74 (61 - 87)	14 (53.8%)	UK	6.0.1
NIMH-IHV (s1)	48	27 (18 - 63)	35 (72.9%)	USA	7.3.2
NIMH-IHV (s2)	54	33 (21 - 71)	48 (88.9%)	USA	7.3.2
NKI	790	39 (6 - 85)	489 (61.9%)	USA	6.0.1
OASIS3	355	67 (42 - 95)	228 (64.2%)	USA	7.3.2
Stanford-CR	40	22 (5 - 27)	19 (47.5%)	USA	7.3.2
UCLH (s1)	28	37 (19 - 64)	16 (57.1%)	UK	7.3.2
UCLH (s3)	67	39 (19 - 65)	43 (64.2%)	UK	7.3.2
Total	3,276	33 (5 - 95)	1,911 (58.3%)		

Table S1.1: Descriptive statistics of each study dataset included in the normative model.

S2 Statistical modelling

S2.1 Model formula

For the normative model, we assume the morphological data, measured in all metrics including cortical thickness, volume, surface area, tension K, shape S, and isometric size I, follow a flexible sinh-arcsinh (shash) distribution, which allows for non-normal modelling of the data in which the first four moments of the distribution can vary as functions of explanatory variables. All metrics are transformed to a logscale before statistical modelling. We use gamlss (`https://www.rdocumentation.org/packages/gamlss/versions/5.4-12/topics/gamlss`) to simultaneously model the parameters (first four moments) of the distribution as response variables of the explanatory variables sex, age, and scanning site. We use the model formulae:

$$
\begin{aligned}
\mu &\sim 1 + sex + s(age) + (1|site) \\
\sigma &\sim 1 + sex + s(age) + (1|site) \\
\nu &\sim 1 + sex + s(age) \\
\tau &\sim 1 + s(age)
\end{aligned}
$$

- The mean (μ) depends on sex (fixed effect), site (random effect), and a smooth function of age.
- The standard deviation (σ) depends on sex (fixed effect), site (random effect), and a smooth function of age.
- The skew (ν) depends on sex (fixed effect) and a smooth function of age.
- The kurtosis (τ) depends on a smooth function of age.

We fitted this model to the normative datasets ($n \sim 3500$) for each metric and cortical region independently.

To test our model formula, we fitted alternative models to our data, changing one model term at a time. This resulted in the comparison of 11 models in total. The model as described above

is considered the default (Model 1 in figures S2.1, S2.2, S2.3). In models 2-11, the formulae of all parameters were kept the same as in model 1, except as described below:

$$\text{Model 2: } \mu \sim 1 + s(age) + (1|site)$$
$$\text{Model 3: } \sigma \sim 1 + s(age) + (1|site)$$
$$\text{Model 4: } \nu \sim 1 + s(age)$$
$$\text{Model 5: } \tau \sim 1 + sex + s(age)$$
$$\text{Model 6: } \nu \sim 1 + sex + s(age) + (1|site)$$
$$\text{Model 7: } \tau \sim 1 + s(age) + (1|site)$$
$$\text{Model 8: } \mu \sim 1 + sex + age + (1|site)$$
$$\text{Model 9: } \sigma \sim 1 + sex + age + (1|site)$$
$$\text{Model 10: } \nu \sim 1 + sex + age$$
$$\text{Model 11: } \tau \sim 1 + age$$

I.e., models 2-5 removed/introduced a sex term to the four parameters, models 6-7 introduced a site effect to the skew and kurtosis, and models 8-11 simplified the smooth function of age to a linear term in all four parameters. We computed AICs (Akaike information criterion) for each model fit in each metric and cortical region, and assessed the optimal model for each metric and region by computing weighted relative AICs (figures S2.1, S2.2, S2.3).

Generally, our model fitted the data better or similarly well as the alternative models. In a subset of cortical regions, model 6 with a random site effect on the skew had a significantly better fit. However, we opted not to include this term in our final model for two reasons: a), to keep the model formula consistent across metrics and regions for comparability, and b) to allow us to apply the normative model to new datasets with small healthy control samples, which would make an accurate estimation of site-specific skew impossible.

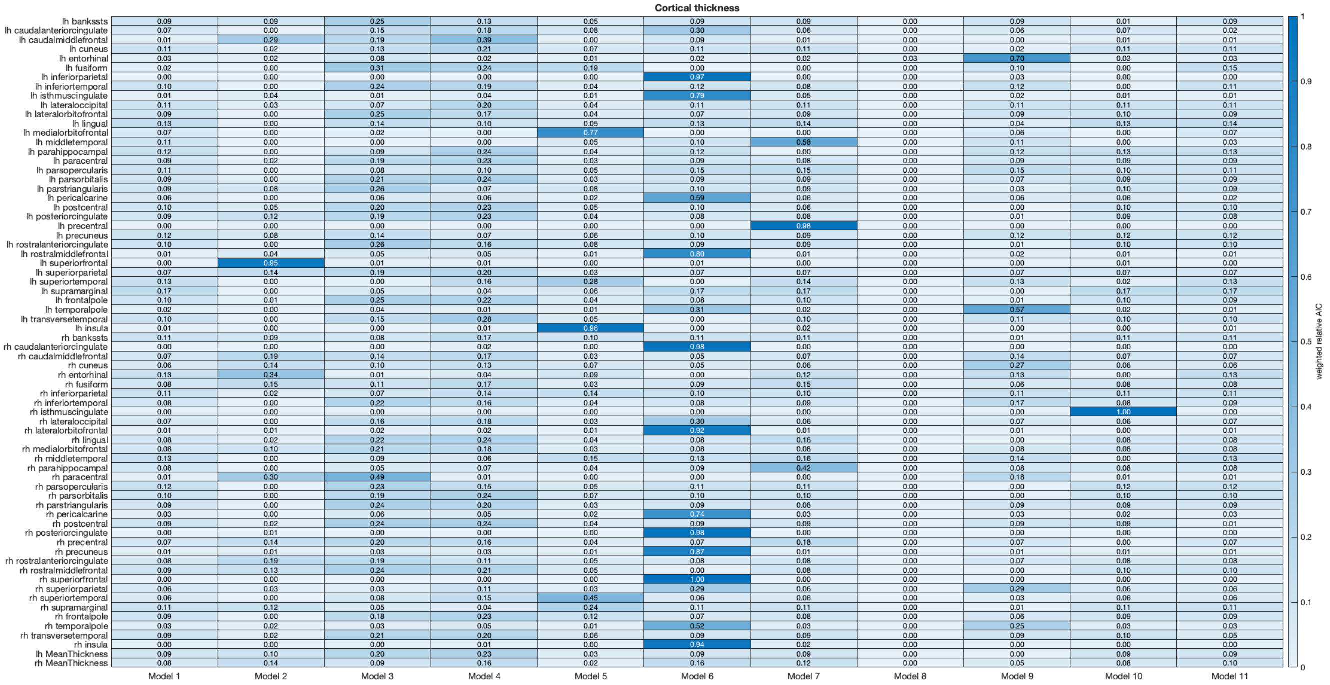

Fig. S2.1: Weighted relative AIC of 11 gamlss models fitted for cortical thickness. Darker colour indicates higher probability that the model is optimal for that metric and region out of the 11 models tested. Rows correspond to 68 regions (Desikan-Killiany atlas) as well as left and right hemisphere mean thickness. Columns correspond to 11 model formulae.

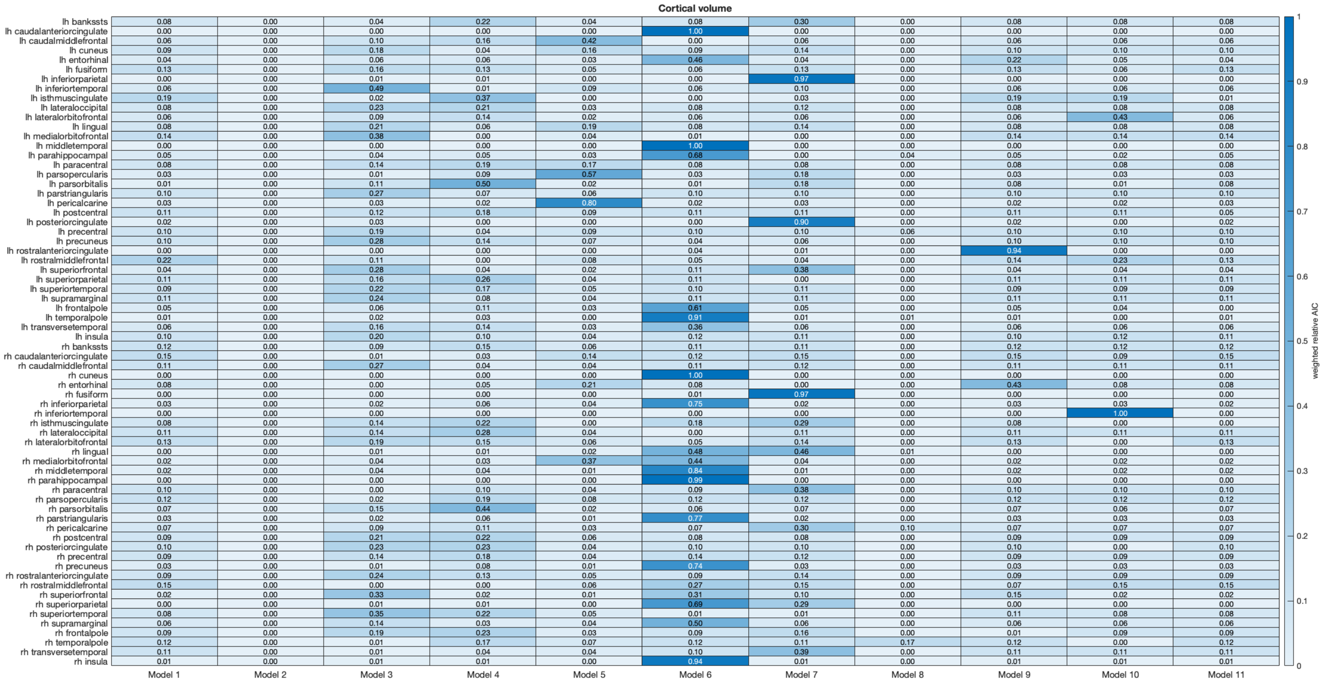

Fig. S2.2: Weighted relative AIC of 11 gamlss models fitted for cortical volume. Darker colour indicates higher probability that the model is optimal for that metric and region out of the 11 models tested. Rows correspond to 68 regions (Desikan-Killiany atlas). Columns correspond to 11 model formulae.

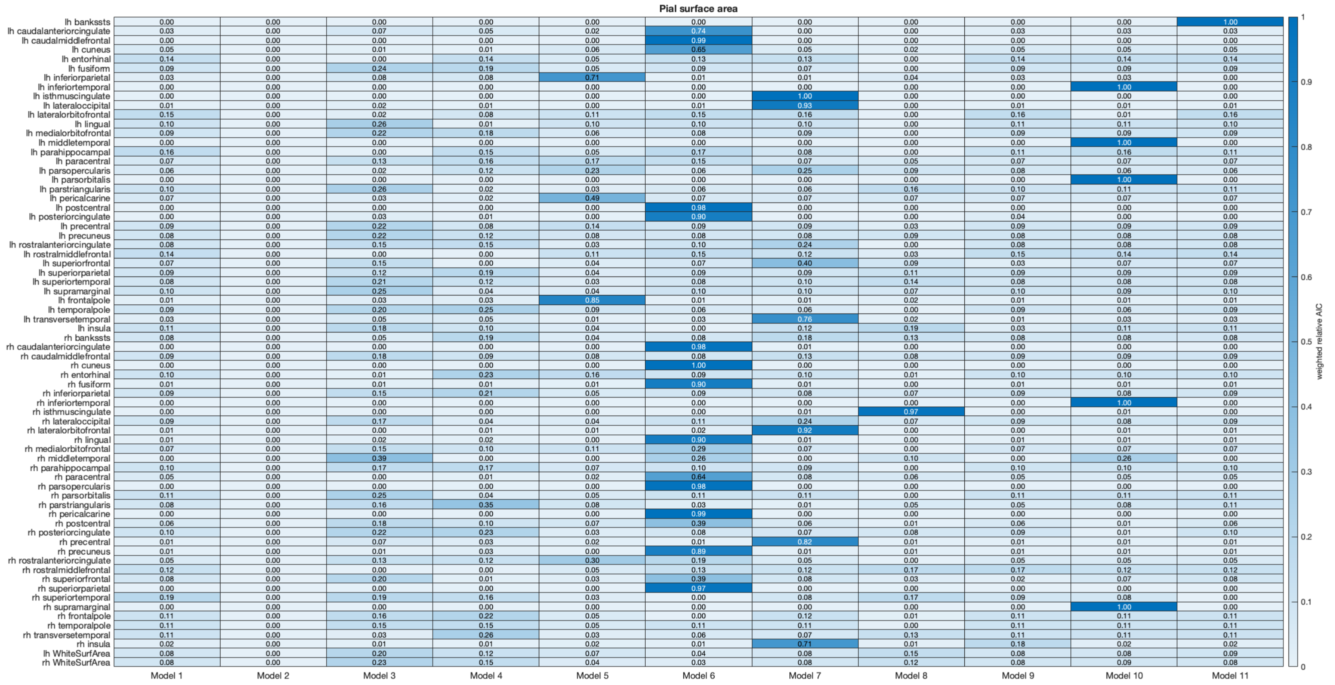

Fig. S2.3: Weighted relative AIC of 11 gamlss models fitted for pial surface area. Darker colour indicates higher probability that the model is optimal for that metric and region out of the 11 models tested. Rows correspond to 68 regions (Desikan-Killiany atlas) as well as left and right hemisphere total surface area. Columns correspond to 11 model formulae.

S2.2 Application to new data

To apply the model to new datasets, we fitted it to the new data assuming it was from one of the scanning sites used for training, to predict model parameters and calculate residuals. This removed site and sex effects from the data. Site-specific mean and variance were then estimated as the mean and variance of the residuals of the new site's healthy controls. The exact steps of this algorithm are described in table S2.4.

Here, steps 5-7 are similar to steps 2-4, but remove the standard deviation from the data rather than the mean. Whilst the mean of a dataset can be estimated reasonably reliably even from small samples, the estimation of standard deviation requires a larger sample. For this reason, we use even small (>10 subjects) healthy control data to estimate the new site's mean (step 3), but require at least 30 subjects for the estimation of the new site's standard deviation (step 6).

To compute centiles, we estimate $\mu'_{hc} = \text{mean}(y_3)$ again from the healthy controls. We then use that site mean, the site standard deviation (σ_{hc}), and the skew and kurtosis from the normative

model (ν_{nm} and τ_{nm}) to get quantiles for each subject and calculate their centiles (Figure S2.5).

Step	Change	Explanation
1	Predict $\mu_{nm}, \sigma_{nm}, \nu_{nm}, \tau_{nm}$ for each subject from normative model.	Predict distribution parameters for each new subject based on their age and sex, but with their site set to one of the normative sites (site A).
2	$y_1 = y - \mu_{nm}$	Calculate residuals for each subject relative to site A by subtracting the predicted μ, which removes age and sex effects and centres the new site around the mean of site A.
3	Estimate $\mu_{hc} = \text{mean}(y_1)$ from healthy controls.	Estimate the mean of the residuals from the healthy controls. This is the site-specific offset of the new site relative to site A.
4	$y_2 = y_1 - \mu_{hc}$	Calculate residuals for each subject relative to their site mean.
5	$y_3 = y_2/\sigma_{nm}$	Divide by the standard deviation predicted by the normative model, to z-score subjects relative to the variance in site A.
6	Estimate $\sigma_{hc} = \text{std}(y_3)$ from healthy controls<br>**or**<br>$\sigma_{hc} = \text{mean}(\sigma_{sites})$	If the new site has more than 30 controls, estimate the standard deviation from the controls relative to site A.<br>**or**<br>If there are fewer than 30 controls, compute the mean of the site-specific standard deviations across normative sites (obtained as their σ coefficients) as an estimate standard deviation instead.
7	$zscore = y_3/\sigma_{hc}$	The data is z-scored by dividing by the site-specific standard deviation.

Table S2.1: Steps to apply the normative model to new data from an unseen scanning site. Here, y is the measured data of one subject in a single cortical region in one metric (e.g. thickness). y_n refers to the data after successive steps of manipulation. $\mu_{nm}, \sigma_{nm}, \nu_{nm}, \tau_{nm}$ are the mean, standard deviation, skew and kurtosis predicted for one subject by the normative model. μ_{hc} and σ_{hc} are the site-specific mean and standard deviation relative to a normative site A.

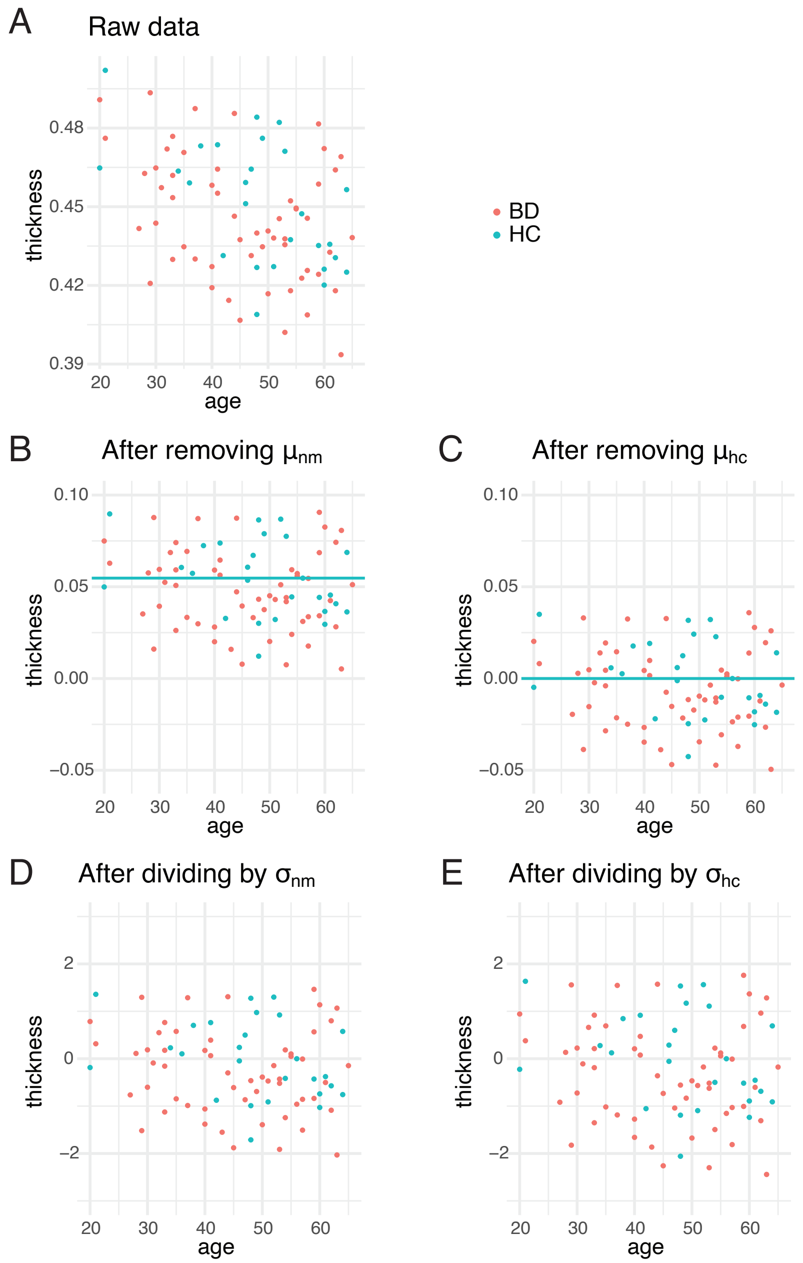

Fig. S2.4: Application of normative model to unseen data. Plots show cortical thickness for one example region. **A** Raw data from new scanning site. **B** Data after removing the mean μ_{nm} estimated from the normative model (step 2.). The blue line indicates the healthy control mean $\mu_h c$. **C** Data after removing the site-specific mean μ_{hc} (step 4.). **D** Data after dividing by the standard deviation σ_{nm} estimated from the normative model (step 5.). **E** Data after dividing by the site-specific standard deviation σ_{hc} (step 7.).

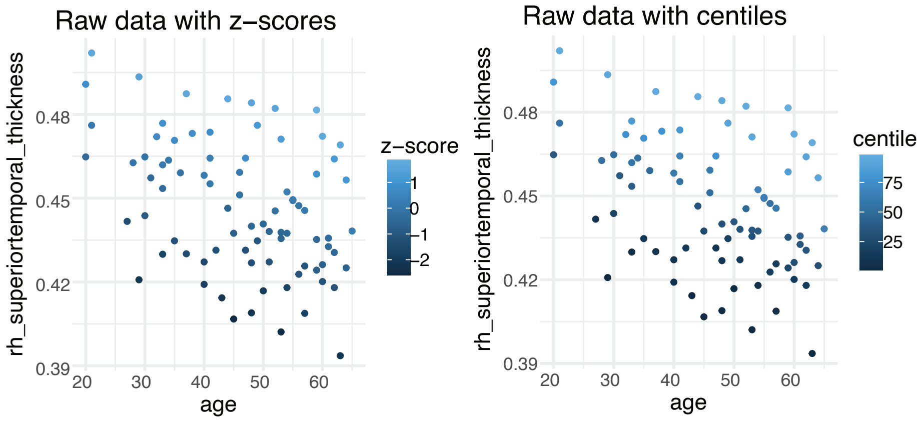

Fig. S2.5: Z-scores and centiles of unseen data.

S3 Assessing model stability through subsampling

To assess the stability of our normative models, we tested correlations between models using a subset of the full data set and the convergence of errors on a random sample of held-out subjects. Figure S3.1 A shows that models trained on sampled subsets of at least 2000 subjects have a high correlation (>99.5%) with the model trained on the full dataset in terms of test set residuals, meaning predictions from the subset-trained model closely match those from the model using all available data. Figure S3.1 B shows that the mean absolute error of predictions on the test set converges when using around 2000 subjects for training, indicating that model performance has little improvement with larger sample sizes. Together, these findings confirm that our model, trained on the full sample (n=3276), is stable.

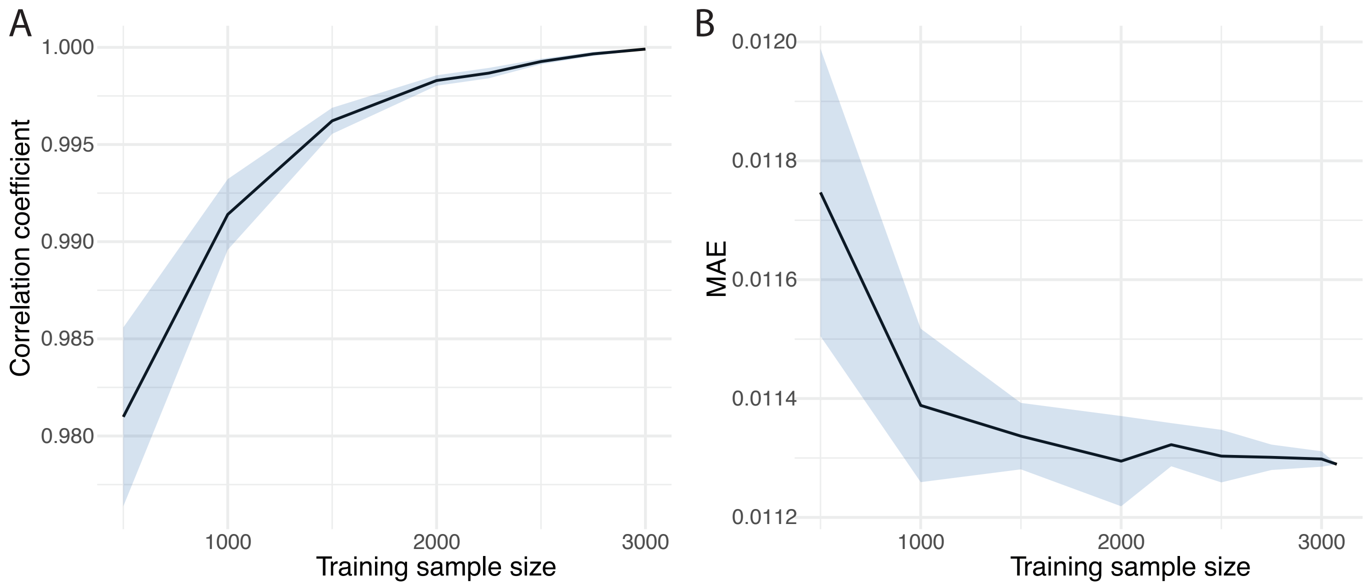

Fig. S3.1: Model stability trained on subsamples of full data. Normative model of right hemisphere cortical thickness (log transformed) retrained on subsamples of the normative data set. 10 samples were repeated at each sample size. 200 subject randomly sampled across sites were held out to assess model similarity and fit across samples. **A)** Pearson correlation coefficient between residuals of 200 held-out subjects using subsampled model and full model (n=3076). Grey shading indicating 95% confidence interval. **B)** Mean absolute error (MAE) of predictions of 200 held-out subject. Grey shading indicates 95% confidence interval.

S4 Validation using mesial temporal lobe epilepsy cohort

S4.1 Comparing group-level cortical thickness abnormalities in mesial temporal lobe epilepsy with ENIGMA findings

We correlated the effect size of the group difference (mTLE versus healthy controls) of cortical thickness of each brain region with the equivalent effect sizes reported in an ENIGMA study (Whelan et al., 2018). This was done for mTLE-left and mTLE-right groups separately. Effect sizes produced by Brain MoNoCle in our sample show agreement (correlation larger than 0.5) with those reported previously, as shown in Figure S4.1. Given that the ENIGMA sample of TLE was much larger, combining data from many more site, and using different data harmonisation methods, we believe that this level of agreement in the resultant effect sizes is remarkable.

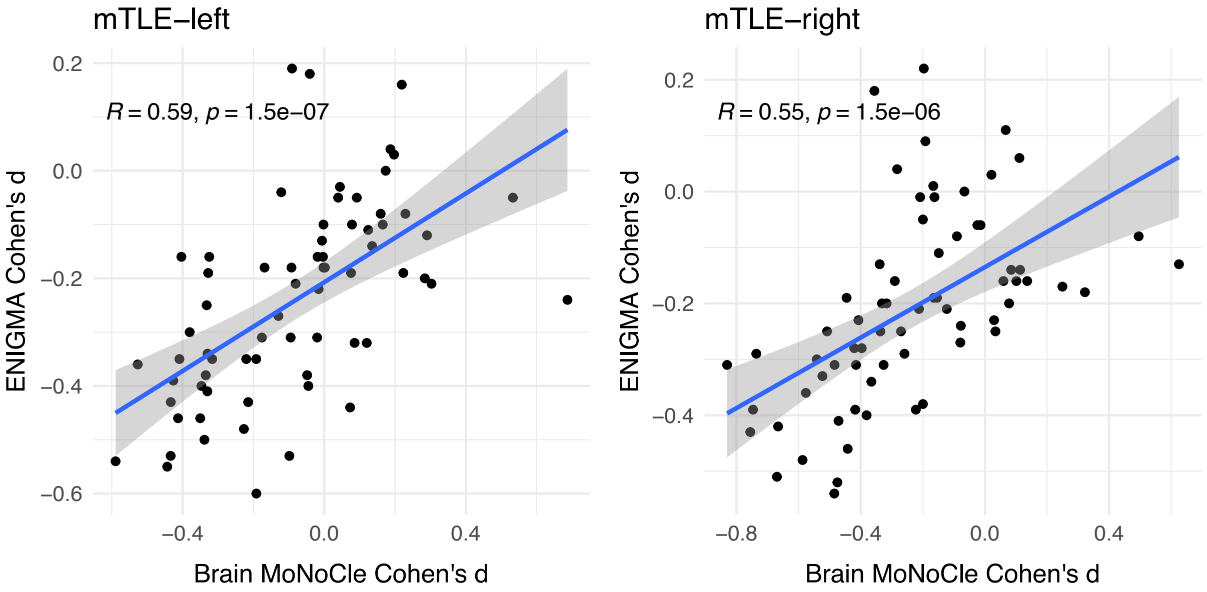

Fig. S4.1: Correlations of cortical thickness abnormalities of each brain region produced by Brain MoNoCle using the IDEAS dataset with equivalent effect sizes reported in the ENIGMA study. Abnormalities were calculated as effect sizes (Cohen's d) when comparing each mTLE group to healthy controls. Left panel=mTLE left-lateralised, right panel=mTLE right-lateralised.

S4.2 Prediction of TLE lateralisation

We validated our normative model by assessing the agreement of a prediction of lateral hemisphere using abnormalities calculated from our normative model with clinical lateralisation in individuals with TLE. For three metrics (cortical thickness, cortical volume, and isometric size (I)), we predicted individuals' side of seizure onset to be the right hemisphere if the difference of left and right hemisphere z-scores (see Figure **??**) was positive, and left if it was negative. Figure S4.2 shows a confusion matrix for each metric. Predictions using cortical volume performed best, achieving an accuracy 0.782.

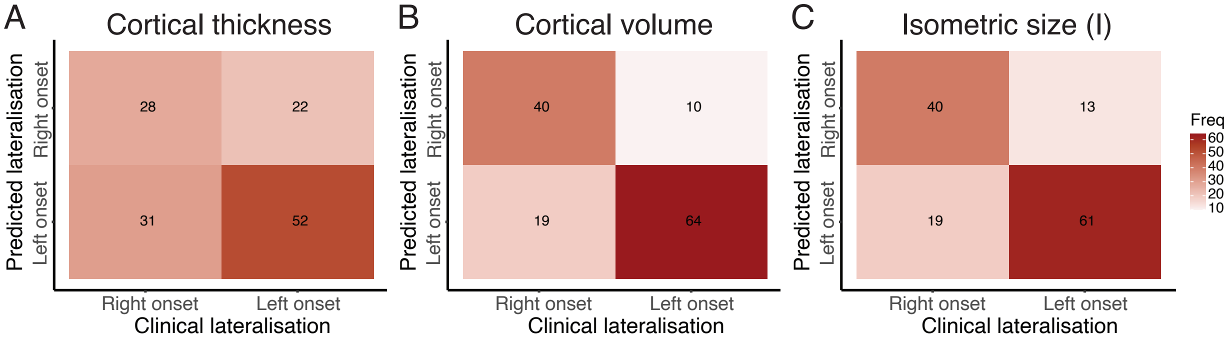

Fig. S4.2: Confusion matrices of predicted TLE lateralisation. Performance of lateralisation prediction based on the sign of the difference of left and right hemisphere z-score. Confusion matrices are shown for metrics average cortical thickness (**A**), cortical volume (**B**), and isomectric size I (**C**).

S5 Comparison of Brain MoNoCle and CentileBrain

We ran data for healthy controls from the IDEAS dataset (n=99) through another normative modelling app, CentileBrain (Ge et al., 2024). We correlated the z-scores produced by CentileBrain and Brain MoNoCle to compare the output from each app. Figure S5.1 illustrates the results of the correlations, which show good agreement between the two apps for most brain regions.

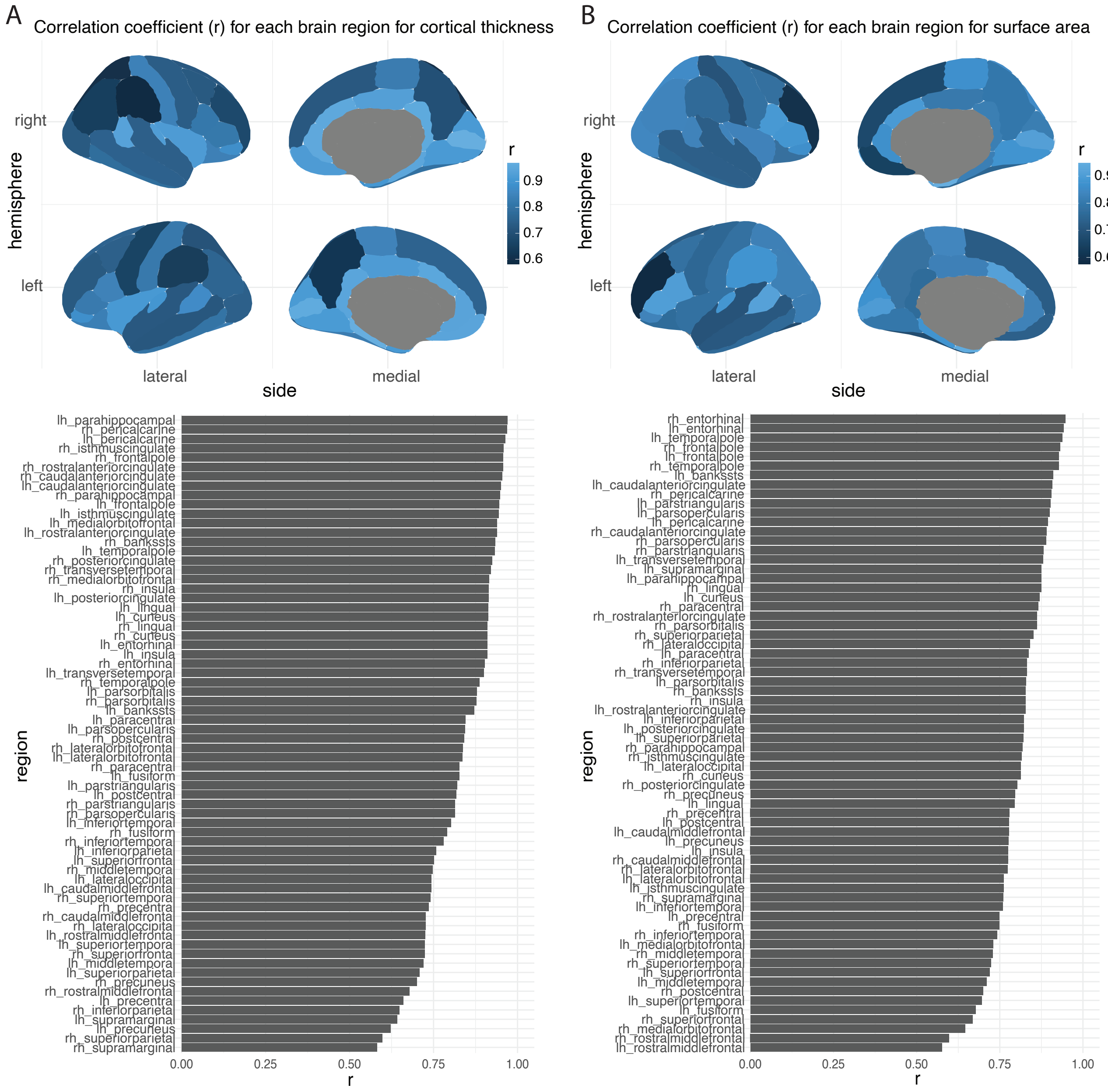

Fig. S5.1: Correlations of z-scores derived from Brain MoNoCle and CentileBrain. Correlation coefficients are presented for each brain region for cortical thickness (**A**) and surface area (**B**).

S6 Additional Acknowledgements

Cambridge Centre for Ageing and Neuroscience (CamCAN). CamCAN funding was provided by the UK Biotechnology and Biological Sciences Research Council (grant number BB/H008217/1), together with support from the UK Medical Research Council and the University of Cambridge, UK.

OASIS-3 provided data: Longitudinal Multimodal Neuroimaging: Principal Investigators: T. Benzinger, D. Marcus, J. Morris; NIH P30 AG066444, P50 AG00561, P30 NS09857781, P01 AG026276, P01 AG003991, R01 AG043434, UL1 TR000448, R01 EB009352. AV-45 doses were provided by Avid Radiopharmaceuticals, a wholly-owned subsidiary of Eli Lilly. HCP data were provided in part by the Human Connectome Project, WU-Minn Consortium (Principal Investigators: David Van Essen and Kamil Ugurbil; 1U54MH091657) funded by the 16 NIH Institutes and Centers that support the NIH Blueprint for Neuroscience Research; and by the McDonnell Center for Systems Neuroscience at Washington University.

The Oregon ADHD1000 raw data is publicly available on the NIMH Data Archive (NDA) under #1938.

We thank the NIMH Office of the Clinical Director, the outpatient behavioral health clinic and NMR center for providing support for the data collection. This work utilized the computational resources of the NIH HPC Biowulf cluster `http://hpc.nih.gov`.

We thank Anna Beres, Koryna Lewandowska, Monika Ostrogorska, Barbara Sikora-Wachowicz, Aleksandra Zyrkowska, Justyna Janik, Kamil Cepuch, and Piotr Faba for assistance with participant recruitment and data collection of chronotype dataset. They are funded by Polish National Science Centre (NCN) Grant 2013/08/M/HS6/00042 and Grant 2013/08/W/NZ3/00700.

Greene data collection was supported by National Institutes of Health Grants K01MH104592 (DJG), K23NS088590, UL1TR000448 (NUFD), R01MH096773, R00MH091238, UL1TR000128, MH110766, U01DA041148 (DAF), P30NS098577 (to the Neuroimaging Informatics and Analysis Center), and U54HD087011 (to the Eunice Kennedy Shriver National Institute Of Child Health & Human Development of the National Institutes of Health to the Intellectual and Developmental Disabilities Research Center at Washington University; BLS), the Tourette Association of America

(DJG), the McDonnell Center for Systems Neuroscience, the Mallinckrodt Institute of Radiology (DJG, NUFD), the DeStefano Family Foundation (DAF), the Jacobs Foundation, and the Child Neurology Foundation (NUFD).

References

Ge, R., Yu, Y., Qi, Y. X., nan Fan, Y., Chen, S., Gao, C., ... Group, E. L. W. (2024). Normative modelling of brain morphometry across the lifespan with centilebrain: algorithm benchmarking and model optimisation. *The Lancet Digital Health*, *6*, e211-e221. doi: 10.1016/S2589-7500(23)00250-9

Whelan, C. D., Altmann, A., Botía, J. A., Jahanshad, N., Hibar, D. P., Absil, J., ... Sisodiya, S. M. (2018). Structural brain abnormalities in the common epilepsies assessed in a worldwide enigma study. *Brain*, *141*, 391-408. doi: 10.1093/BRAIN/AWX341
